# Evidence on Efficacy and Safety of Chinese Medicines Combined Western Medicines Treatment for Breast Cancer With Endocrine Therapy

**DOI:** 10.3389/fonc.2021.661925

**Published:** 2021-06-21

**Authors:** Lu Li, Rongyun Wang, Aolin Zhang, Ling Wang, Qianwen Ge, Yuan Liu, Tianhui Chen, Chi Chiu Wang, Ping Chung Leung, Qiuhua Sun, Xiaohui Fan

**Affiliations:** ^1^ College of Pharmaceutical Sciences, Zhejiang University, Hangzhou, China; ^2^ Innovation Center in Zhejiang University, State Key Laboratory of Component-Based Chinese Medicine, Hangzhou, China; ^3^ Department of Obstetrics & Gyneacology, The Chinese University of Hong Kong, Hong Kong, Hong Kong; ^4^ Institute of Chinese Medicine, The Chinese University of Hong Kong, Hong Kong, Hong Kong; ^5^ School of Nursing, Zhejiang Chinese Medical University, Hangzhou, China; ^6^ The First College of Clinical Medicine, Zhejiang Chinese Medical University, Hangzhou, China; ^7^ The First College of Clinical Medicine, Lanzhou University, Lanzhou, China; ^8^ Department of Cancer Prevention/Experimental Research Center, Cancer Hospital of the University of Chinese Academy of Sciences (Zhejiang Cancer Hospital), Institute of Basic Medicine and Cancer (IBMC), Chinese Academy of Sciences, Hangzhou, China

**Keywords:** breast cancer, Chinese medicines combined western medicines, efficacy, endocrine therapy, safety

## Abstract

**Background:**

Breast cancer, a malignant disorder, occurs in epithelial tissue of the breast glands and ducts. Endocrine therapy is commonly applied as an important adjuvant treatment for breast cancer, but it usually induces a variety of side effects. Chinese Medicines (CM) has therapeutic effect on reducing adverse effects of the endocrine therapy in many clinical studies. But strong evidence is still limited on the efficacy and safety of CM combined western medicines (CM-WM) for breast cancer.

**Objective:**

To study the efficacy and safety of CM-WM as an adjuvant treatment for reducing side effects induced by endocrine therapy in breast cancer patients.

**Method:**

We searched relevant clinical studies in PubMed and the Chinese National Knowledge Infrastructure (CNKI) databases up to February 28, 2021 and only Randomized Controlled Trials (RCTs) were included. There were no limitations on the languages. We extracted data from the included RCTs, assessed study quality, conducted meta-analyses by RevMan 5.4 and compared the pooled Risk Ratios (RR) or Mean Difference (MD) with 95% CIs.

**Results:**

In total 28 trials involving 1,926 participants were included. Six RCTs compared CM-WM with CM placebo-WM, while 22 RCTs compared CM-WM with WM alone. No study compared CM-WM with no treatment. Meta-analysis showed that CM-WM treatment significantly improved quality of life (MD = 0.73, 95% CI = 0.11–1.35, *P* = 0.02) when compared with CM placebo-WM treatment. When compared with WM treatment alone, CM-WM treatment significantly improved bone mineral density (MD = 0.24, 95% CI = 0.13–0.35, P <0.0001), TCM syndrome score (MD = −5.39, 95% CI = −8.81 to −1.97, *P* = 0.0002), Kupperman Scale (MD = 0.24, 95% CI = −2.76 to −1.94, *P <*0.0001), Karnofsky Performance Scale (MD = 3.76, 95% CI = 1.64–5.88, *P* = 0.0005), quality of life (MD = 3.01, 95% CI = 1.00–5.02, *P* = 0.003), and pain relief (MD = 2.10, 95% CI = 0.72–3.48, *P <*0.0001). Compared with WM, CM-WM significantly decreased incidence of TCM symptoms (nausea, vomiting, fatigue, etc.) (RR = 1.60, 95% CI = 1.40–1.84, *P <*0.0001). For safety, serum calcium, estradiol, ALP, and blood CD3, CD4 and CD8 counts were not significantly difference between two treatments (*P >*0.05). Serious side effects or reactions were not reported in all included studies.

**Conclusion:**

The adjunctive use of CM reduced the endocrine therapy associated adverse events, including bone mineral density loss, perimenopausal symptoms, poor quality of life, pain and impaired immune function. But large-scale and high quality RCTs are needed to support the application of CM-WM therapy.

## Background

Breast cancer, a malignant disorder, occurs in the epithelial tissue of breast glands and ducts (1). In recent years, the incidence rate of breast cancer is slightly increases at 0.4%/year (2). According to the estimation of American Cancer Society (ACS) for 2019 in the United States (2), more than 0.2 million new invasive breast cancer will be diagnosed, while about 41,760 women will die from the cancer. The chance of any woman dying from breast cancer is around one in 38 (2.6%) (3). In China, breast cancer is the second common cancer in female, of which the incidence is about 169,000 every year (4). Due to early diagnosis of breast cancer by increased awareness, early screening improved treatment response, and mortality of patients decreased 40% in the past 30 years (2, 3).

Nowadays, surgery, chemotherapy, endocrine therapy, immunotherapy, radiation and targeted therapies are acknowledged as common treatments in breast cancer (4–7). As cancer cells may not be completely removed by surgery or have already spread unnoticeably before treatment, endocrine therapy as an adjuvant treatment is necessary and commonly applied (8, 9). Endocrine therapy is to change the endocrine environment needed for hormone-dependent tumor growth by inhibiting or interfering the process of binding of hormone receptor, for instance estrogen receptor in breast cancer, so as to restrain the proliferation of tumor cells. The mechanisms of endocrine therapy in breast cancer include inhibiting the synthesis of estrogen, reducing the level of estrogen, blocking the binding of estrogen and its receptors, and reducing the activity of receptors, etc. (10). It can reduce the recurrence of breast cancer and improve the survival rate of patients (10, 11). Currently, commonly used endocrine therapy drugs include Tamoxifen, Aromatase Inhibitors (Letrozole, Anastrozole), etc. (12, 13). They can eliminate malignant tumor cells, but can also lead to adverse outcomes that negatively affect compliance, especially on bone health and perimenopausal symptoms (14, 15). Therefore, an intervention to reduce the side effects of endocrine therapy as well as to increase the tolerance and well-being of cancer patients is necessary.

Complementary alternative medicine (CAM) has been widely used for a long time for cancer treatment. As an important part of CAM, Traditional Chinese Medicine (TCM) has formed its own unique system of theory, diagnosis and treatment modality in Asian countries, especially in China. Chinese Medicine (CM), as one common approach of TCM, has been increasingly used in the last decades, especially as a complementary treatment to endocrine therapy. It can improve clinical symptoms, relieve or reduce adverse outcomes due to endocrine therapy and prolong patients’ survival time. Many clinical studies suggested that the therapeutic effects of CM for cancer treatment may work in two aspects. Firstly, it can improve the function of the immune system and prevent tumor recurrence and metastasis. Secondly, it can reduce or prevent the toxicity of conventional anti-cancer drugs, while improve their therapeutic effects. However, systematic review to evaluate the efficacy and safety of CM as an adjuvant treatment in breast cancer patients is still lacking.

## Methodology

### Criteria for Inclusion

#### Subjects

1. Postoperative breast cancer patients under treatment of endocrine therapy;
2. Only patients with primary tumors were included;
3. There were no contraindications to endocrine therapy;
4. There were no severe diseases found in other systems and organs;
5. The patients did not have other untreated malignant disorders simultaneously;
6. The patients were tolerant to the endocrine therapy with life expectancy at least six months, and;

#### Types of Studies

1. Only RCTs were included;
2. Western Medicine (WM) was any endocrine therapy drug;
3. The baseline was comparable.

#### Interventions

1. CM combined with WM (CM-WM) versus CM placebo combined with WM (CM placebo-WM);
2. CM combined with WM (CM-WM) versus no treatment;
3. CM combined with WM (CM-WM) versus WM alone.

### Criteria for Exclusion

1. Diagnostic criteria are unclear;
2. Allergic to the endocrine therapy drug;
3. With non-primary breast cancer or complicated with other malignancies;
4. With serious diseases in major organs such as heart, liver, brain, kidney and other systemic diseases;
5. Shedding cases were excluded, for example, poor compliance subjects, severe adverse events, complications or other situations that cannot continue the treatment, request to quit the study, etc.

### Literature Search

#### Database

We searched systematically all the potentially relevant publications related to CM-WM for breast cancer in PubMed and CNKI databases. All databases were searched from 1st June, 1986 to 28th February, 2021.

#### Search Strategy

The keywords used for PubMed, Medline and Cochrane database search were as follows: [(breast cancer) OR (mammary cancer) OR (breast tumor)] AND [(Chinese medicine) OR (traditional Chinese medicine) OR (Chinese medicines combined western medicines) OR (integrative medicine) OR (herbal medicine)]. Chinese Pinyin and character searches were applied in the CNKI database. There were no limitations on the language.

#### Data Extraction

Two authors independently checked all identified clinical trials (firstly titles and abstracts, then full-texts), basic on the pre-designed standard data extraction form to remove improper studies according to the inclusion and exclusion criteria. Full-texts of these studies were further checked. A third author made the consensus when there was any nonconformity. Authors extracted information from all included RCTs, including publication year, study design, study size, baseline data, randomization methods, therapeutic results, adverse events, etc.

#### Quality Assessment

The assessment criteria of methodological quality in this review were designed in accordance with the Cochrane Handbook for Systematic Reviews of Interventions (16). Baseline information, randomization, allocation concealment, blinding, patient withdrawal or loss in follow-up, were recorded and summarized.

#### Data Analysis

The data were processed and analyzed according to the Cochrane Handbook (8), by Cochrane recommended software Review Manager (version 5.4). As to dichotomous and continuous data, pooled RR (Risk Ratio) and MD (Mean Difference) were applied with 95% CIs (Confidence Intervals), respectively. Forest charts were conducted for heterogeneity test, sensitivity analysis and bias report. We defined statistical significance by p value <0.05.

Different effect models and heterogeneity analyses were applied according to the Cochrane Handbook. If the included trials reported the same treatment effects, a fixed-effect model was applied to combine and compare the extracted data. When heterogeneity analysis I^2^ >50% was found in the fixed-effect model, a random-effect model would be applied. When MD data was equivalent to RR, we also used a random-effect model.

## Results

### Literature Search Results

**Figure 1:**
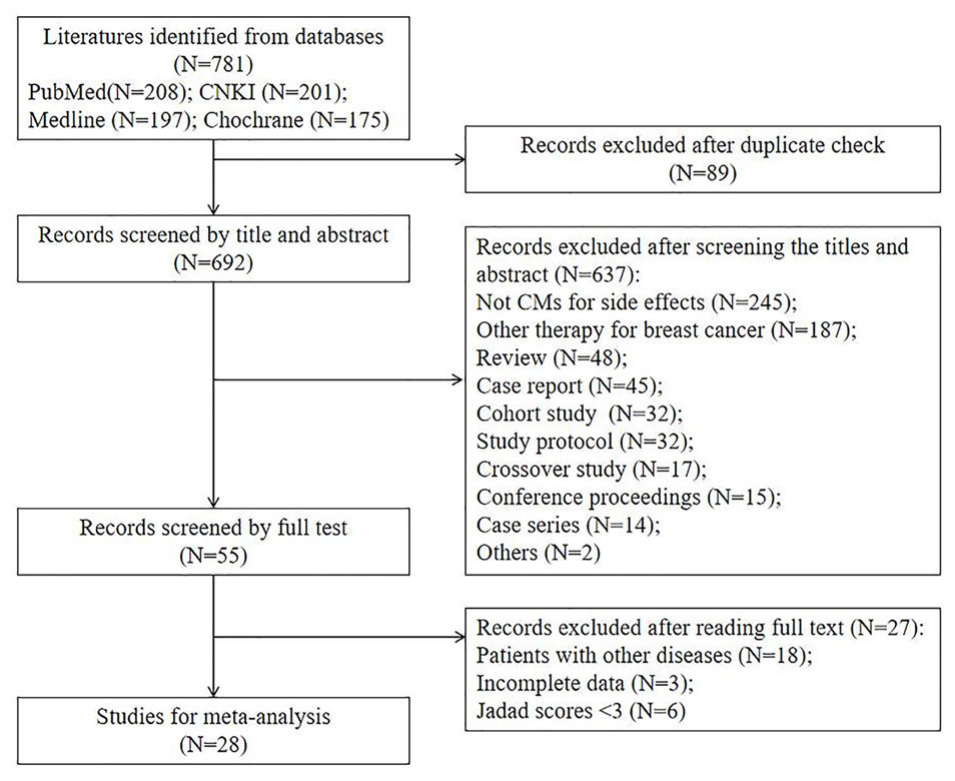
Study inclusion and exclusion flow diagram.

From our literature search, 781 clinical trials were identified. About 692 trials were excluded initially after checking the duplicated publications and reading the study title and abstract. After reviewing the full texts of the remaining 55 studies, we further excluded 27 trials and their exclusion reasons are listed in **Figure 1**. At the end, 28 studies were included for meta-analysis (17–44). We summarized and reported the details of study screening and selection as in **Figure 1**.

### Characteristics and Quality of Included Clinical Trials

The data of 28 RCTs involving 1,926 patients were analyzed, and their characteristics are summarized in **Table 1**. There were 971 patients in the study group (treated by either CM-WM or CM placebo-WM), while 955 in the control group (treated by western medicines only, WM). No study compared CM-WM with any treatment.

**Table 1:** Summary of Characteristics.

Study ID	No. of participants		Age	Stage	Intervention		Duration	Outcomes
	Treatment	Control	(mean ± SD)		Treatment	Control		
Sun (17)	37	36	T: 45.9 ± 5.1	NA*	Tamoxifen, 10 mg, qd, po; Shugan Liangxue Decoction, 30 ml, tid, po	Tamoxifen, 10 mg, qd, po; CM placebo, 30 ml, tid, po	21 days	Efficacy of TCM symptoms Adverse
			C: 46.4 ± 4.1					
Chen (18)	34	30	42 (28–45)	I–III	Tamoxifen, 10 mg, bid, po; Yupingfeng granules, 5g, tid, po	Tamoxifen, 10 mg, bid, po	Not reported	Efficacy of TCM symptoms
Bian (19)	40	40	40–60	I–III	Tamoxifen, 10 mg, bid, po; Shugan Tiaoyinyang Decoction, 200 ml, bid, po	Tamoxifen, 10 mg, bid, po	2 months	KPS Scale; TCM syndrome score kupperman score
Xie (20)	30	30	T: 30–39 y (1);	NA*	Tamoxifen, 10 mg, bid, po; Yishen Chenqian Decoction, 100 ml, bid, po	Tamoxifen, 10 mg, bid, po	3 months	kupperman score KPS E2 Immune function
			40–49 y (26); 50–55 y (3)					
			C: 30–39 y (3);					
			40–49 y (23); 50–55 y (4)					
Li (21)	21	16	60 (36–67)	I–III	AIs, po; Shugan Jiangu granules, 6 g, bid, po	AIs, po	6 months	BMD
Sun (22)	31	31	T: 54.83 ± 6.76	I–IIIa	Letrozole, 2.5 mg, qd, po; calcium carbonate d3, 1 tablet, qd, po; Zuogui Pill, 200 ml, bid, po	Letrozole, 2.5 mg, qd, po; calcium carbonate d3, 1 tablet, qd, po	6 months	BMD Blood calcium, Efficacy of TCM symptoms TCM syndrome scores Quality of life safety assessment
			C: 55.74 ± 5.74					
Ni (23)	25	25	T: 60.85 ± 9.03	I–III	Letrozole, 2.5 mg, qd, po; fine-tune Decoction, 100 ml, bid	Letrozole, 2.5mg, qd, po;	12 months	TCM syndrome scores KPS score Sex hormone level Safety assessment BMD ALP Calcium concentration
			C: 59.95 ± 8.11					
Kong (24)	31	30	T: 59.43 ± 3.37	I–III	Anastrozole, 1.2 g, tid, po; calcium carbonate d3, 0.6 g, qd, po JTG Capsule, 1.2 g, tid, po	Anastrozole, 1.2 g, tid, po; calcium carbonate d3, 0.6 g, qd, po	6 months	BMD Efficacy of TCM symptoms ALP Calcium concentration
			C: 60.07 ± 2.48					
Zhang (25)	42	41	48–75 (median 62)	NA*	Diphosphate, 4 mg, once in 6 months, ivgtt, guzhishusong paste, 20 g, po	Diphosphate, 4 mg, once in 6 months, ivgtt	3 months	BMD
Lu (26)	35	35	T: 58.34 ± 10.63	I–IIIa	AIs, po; Shuanghuang Yigu Decoction, 200 ml, tid	AIs, po	3 months	VAS Score BMD ALP E2
			C: 62.71 ± 11.24					
Liu (27)	32	30	40–60	NA*	Tamoxifen, 10mg, bid, po; Sanhuang Decoction, 100ml, bid, po	Tamoxifen, 10 mg, bid, po	6 months	kupperman score Estradiol Safety assessment
Li (28)	35	35	T: 55 (median)	I–III	AIs, po Calcium carbonate tablets, 600 mg, qd, po; Tiger bone powder, 1.2 g, qd, po	AIs, po Calcium carbonate tablets, 600 mg, qd, po; CM placebo, 1.2 g, qd, po	12 weeks	VAS FACT-B Sex hormone level
			C: 52 (median)					
Peng (29)	42	42	T: 57.3 ± 6.4	I–III	AIs, po; YSJG granules, 200 ml, bid, po; calcium carbonate tablets + vitamin D3, 2 tablet, qd, po	AIs, po; CM placebo, 200ml, bid, po; calcium carbonate tablets +vitamin D3, 2 tablet, qd, po	12 weeks	FACT-B. BMD Safety assessments
			C: 59.8 ± 8.0					
Huang (30)	30	30	T: 50.73 ± 5.21	I–III	Letrozole, 2.5 mg, qd, po; Vitamin D calcium, 600 mg, qd, po; Zishuipeitu Decoction, 10 g, bid,po	Letrozole, 2.5 mg, qd, po; Vitamin D calcium, 600 mg, qd, po;	6 months	Efficacy of TCM symptoms Serum calcium index ALP BMD TCM syndrome scores E2 safety
			C: 51.54 ± 6.89					
Wu (31)	63	63	T: 44.02 ± 5.16	I–III	Tamoxifen, 10 mg, bid, po; cantharidin capsule, 0.75 g, bid, po	Tamoxifen, 10 mg, bid, po;	3 months	FACT-B Immune function Adverse
			C: 44.16 ± 5.19					
Yin (32)	58	58	T: 58.86 ± 7.047	NA*	AIs, po; Nourishing kidney and strong bone prescription, 20 ml, bid, po	AIs, po	3 months	BMD Safety assessment
			C: 58.25 ± 5.973					
Luo (33)	33	33	T: 56.03 ± 6.789	I–IV	Zoledronic, 4 mg, once in 6 months, ivgtt; Jiangu Gao Decoction, 20 g, tid, po	Zoledronic, 4 mg, once in 6 months, ivgtt	6 months	BMD TCM syndrome scores Quality of life Safety assessment Efficacy of TCM symptoms
			C: 57.85 ± 7.620					
Xu (34)	65	65	T: 55.61 ± 4.03	I–IIIa	AIs, po; calcium carbonate d3, 0.6g, qd, po; Modified Sangu Decoction, 200 ml, bid, po	AIs, po; calcium carbonate d3, 0.6 g, qd, po;	12 months	BMD Safety assessment
			C: 57.11 ± 4.89					
Wang (35)	17	15	T: 53.80 ± 7.04	I–III	Tamoxifen, 10 mg, bid, po. CM Decoction, 200 m, bid, po	Tamoxifen, 10 mg, bid, po. CM placebo, 200 m, bid, po	6 months	TCM syndrome score Efficacy of TCM symptoms Immune function Quality of life Safety assessment
			C: 51.85 ± 7.84					
Zhou (36)	54	54	T: 58.18 ± 3.62	I–III	Exemestane, 25 mg, qd, po/Letrozole, 2.5 mg, qd, po/Tamoxifen, 10 mg, bid, po; Yiqi Wenyang Decoction, 200 ml, bid, po	Exemestane, 25 mg qd, po/Letrozole, 2.5 mg, qd, po/Tamoxifen, 10 mg, bid, po	6 months	Quality of life: (QLQ-BR53)
			C: 57.27 ± 10.76					
Hu (37)	20	20	T: 62.35 ± 8.65	I–III	AIs, po; Jianpibushenhuoxue Decoction, po	AIs, po	6 months	BMD E2 VAS TCM syndrome score Kupperman scale ALP
			C: 61.85 ± 7.23					
Cai (38)	25	23	T: 55(26–75)	I–IV	endocrine therapy drug, po; Chaiguilongmu granules, 200 ml, bid, po	endocrine therapy drug, po; Placebo granules, 200 ml, bid, po	30 days	TCM syndrome Score Safety
			C: 55(33–77)					
Liu (39)	24	24	T: 58.46 ± 7.64	NA*	endocrine therapy drug, po; Biejia Jieyu decoction, 150 ml, bid, po	endocrine therapy drug, po	2 weeks	TCM syndrome scores Efficacy of TCM symptoms KPS score Safety Assessment
			C: 59.17 ± 6.29					
Xiao (40)	26	27	T: 60.8 ± 8.7	I–II	Letrozole, 2.5 mg, qd, po; Modified Zhibai Dihuang Decoction, 200 ml, bid, po	Letrozole, 2.5 mg, qd, po	8 weeks	Kupperman score FACT-B score Adverse TCM syndrome score Estradiol
			C: 62.1 ± 9.4					
Tan (41)	30	30	T: 54.5 ± 11.23	I–IV	AIs, po; Alprazdam, 0.4 mg, qd, po; Jianpi Zishen Decoction, 200 ml, bid, po	AIs, po; Alprazdam, 0.4 mg, qd, po	14 days	TCM syndrome scores Efficacy of TCM symptoms Safety assessment
			C: 54.1 ± 13.23					
Liu (42)	28	29	T: 56.64 ± 8.89	I–IV	Third-generation aromatase inhibitors, po; Wenyang Yiqi Decoction, 200 ml, bid, po	Third-generation aromatase inhibitors, po; CM placebo, 200 ml, bid, po	90 days	Efficacy of TCM symptoms FACT-B score TCM syndrome scores
			C: 56.55 ± 6.49					
Du (43)	29	29	T: 44.10 ± 5.72	NA*	Tamoxifen, 10mg, bid, po; Yisheng Hehuo Decoction, 200ml, bid	Tamoxifen, 10 mg, bid, po	84 days	TCM syndrome scores Kupperman score KPS score FACT-B score Serum tumor markers Safety assessment
			C: 42.76 ± 6.11					
Xu (44)	34	34	T: 59.06 ± 7.16	NA*	AIs, po; Calcium tablets, 1 tablet, bid, po; Calcitriol, 1 tablet, bid, po; Zishui Tongluo Decoction, 150 ml, tid	AIs, po; Calcium tablets, 1 tablet, bid, po; Calcitriol, 1 tablet, bid, po	2 weeks	TCM syndrome scores Quality of life (QOL) Safety and adverse BMD Efficacy of TCM symptoms ALP
			C: 58.65 ± 6.174					

*NA, Not available.

Baseline demographics and clinical characteristics were comparable among these trials. No significant differences were found in age at diagnosis, body mass index (BMI), familial history of breast cancer, fertility status, histological type, TNM classification and stage, nuclear grading, hormone receptors status including estrogen receptor (ER), progesterone receptor (PR) and Her2/neu expression and other baseline information between these two groups (P >0.05).

Some six RCTs compared CM-WM with CM placebo-WM, while 22 RCTs compared CM-WM with WM alone. Detailed information is summarized in **Table 1**.

**Table 2** showed the quality assessment of the included clinical trials. Randomization was reported and applied in all included RCTs. Among of them, 14 trials used random number table method and one trial used digital grouping, and five trials applied double-blinding. None of these trials mentioned allocation concealment. The pharmacological characteristics evaluated in each study are recorded in the **Supplementary Table**.

**Table 2:** Quality Assessment.

Study ID	Randomization	Allocation Concealment	Inclusion Criteria	Blinding	Drop-off (%)
Sun (17)	random number table	Not reported	Comparable (*P > *0.05)	Double-blinding	0
Chen (18)	Randomized	Not reported	Comparable (*P > *0.05)	Double-blinding	0
Bian (19)	random number table	Not reported	Comparable (P > 0.08)	Not reported	0
Xie (20)	random number table	Not reported	Comparable (P > 0.05)	Not reported	0
Li (21)	random number table	Not reported	Comparable (P > 0.09)	Not reported	2.63
Sun (22)	randomized	Not reported	Comparable (P > 0.05)	Not reported	0
Ni (23)	unclear	Not reported	Comparable (P > 0.05)	Not reported	18.00
Kong (24)	random number table	Not reported	Comparable (P > 0.10)	Not reported	0
Zhang (25)	randomized	Not reported	Comparable (P > 0.05)	Not reported	0
Lu (26)	random number table	Not reported	Comparable (P > 0.07)	Not reported	0
Liu (27)	randomized	Not reported	Comparable (P > 0.05)	Not reported	0
Li (28)	random number table	Not reported	Comparable (P > 0.05)	Double-blinding	0
Peng (29)	randomized	Not reported	Comparable (P > 0.05)	Not reported	3.75
Huang (30)	digital grouping methods	Not reported	Comparable (P > 0.05)	Not reported	12.98
Wu (31)	random number table	Not reported	Comparable (P > 0.05)	Not reported	0
Yin (32)	random number table	Not reported	Comparable (P > 0.05)	Not reported	0
Luo (33)	randomized	Not reported	Comparable (P > 0.05)	Not reported	0
Xu (34)	randomized	Not reported	Comparable (P > 0.05)	Not reported	0
Wang (35)	randomized	Not reported	Comparable (P > 0.05)	Double-blinding	0
Zhou (36)	random number table	Not reported	Comparable (P > 0.05)	Not reported	4.00
Hu (37)	random number table	Not reported	Comparable (P > 0.11)	Not reported	0
Cai (38)	random number table	Not reported	Comparable (P > 0.12)	Double-blinding	4.00
Liu (39)	random number table	Not reported	Comparable (P > 0.05)	Not reported	0
Xiao (40)	random number table	Not reported	Comparable (P > 0.05)	Not reported	0
Tan (41)	randomized	Not reported	Comparable (P > 0.05)	Not reported	0
Liu (42)	randomized	Not reported	Comparable (P > 0.05)	Double-blinding	3.00
Du (43)	randomized	Not reported	Comparable (P > 0.05)	Not reported	0
Xu (44)	unclear	Not reported	Comparable (P > 0.05)	Not reported	5.56

### Results on Efficacy and Safety

#### Efficacy

##### Bone Mineral Density (BMD)

**Figure 2:**
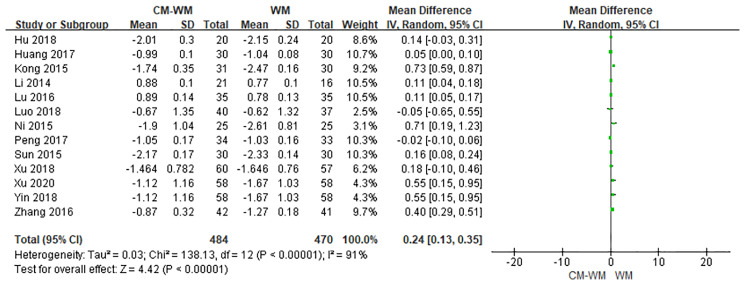
Meta-analyses on BMD.

Thirteen trials recorded bone mineral density after the treatment (21–26, 29, 30, 33, 34, 37, 44). As indicated in the forest plot, the mean bone mineral density (BMD) were significantly higher in CM-WM treatment group compared with WM group (*P <*0.0001, MD = 0.24, 95% CI = 0.13–0.35, **Figure 2**).

##### Menopausal-Like Symptoms

**Figure 3:**
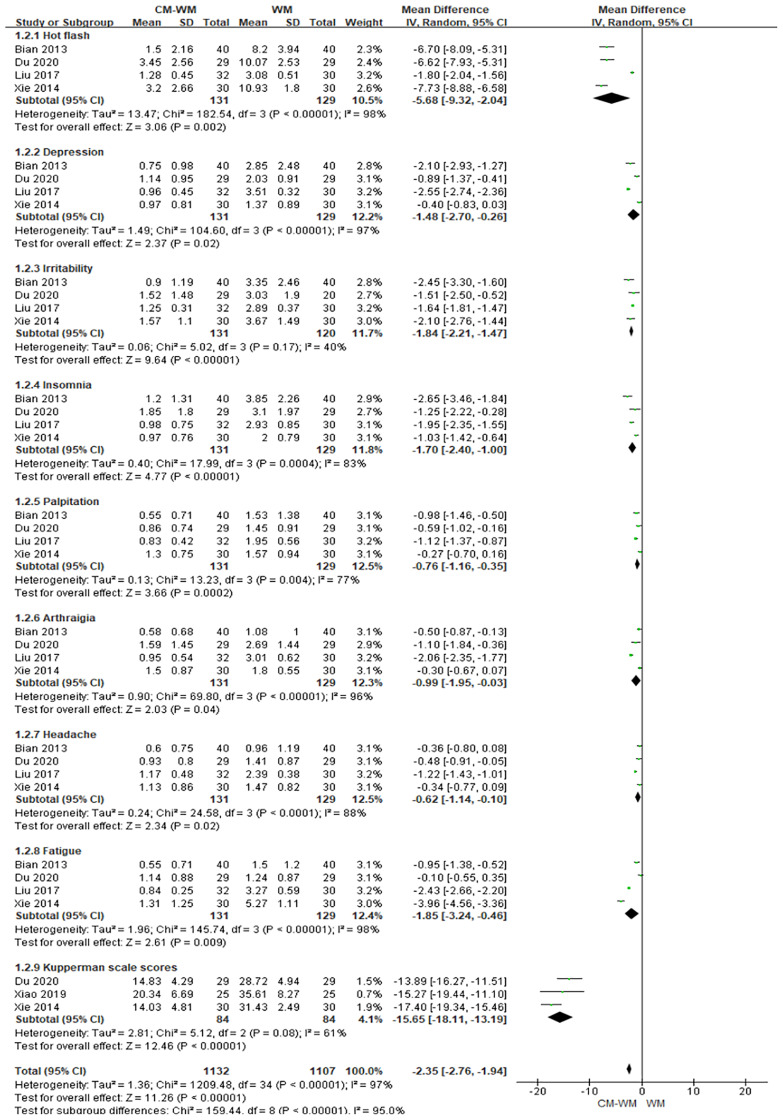
Meta-analyses on Kupperman scales.

Five trials (19, 20, 27, 40, 43) used Kupperman scales to assess the menopausal-like symptoms. As indicated in the forest plot, the mean Kupperman scales was significantly lower in CM-WM group compared with WM group (*P <*0.001, MD = −2.35, 95% CI = −2.76 to −1.94, **Figure 3**).

##### Quality of Life

**Figure 4:**
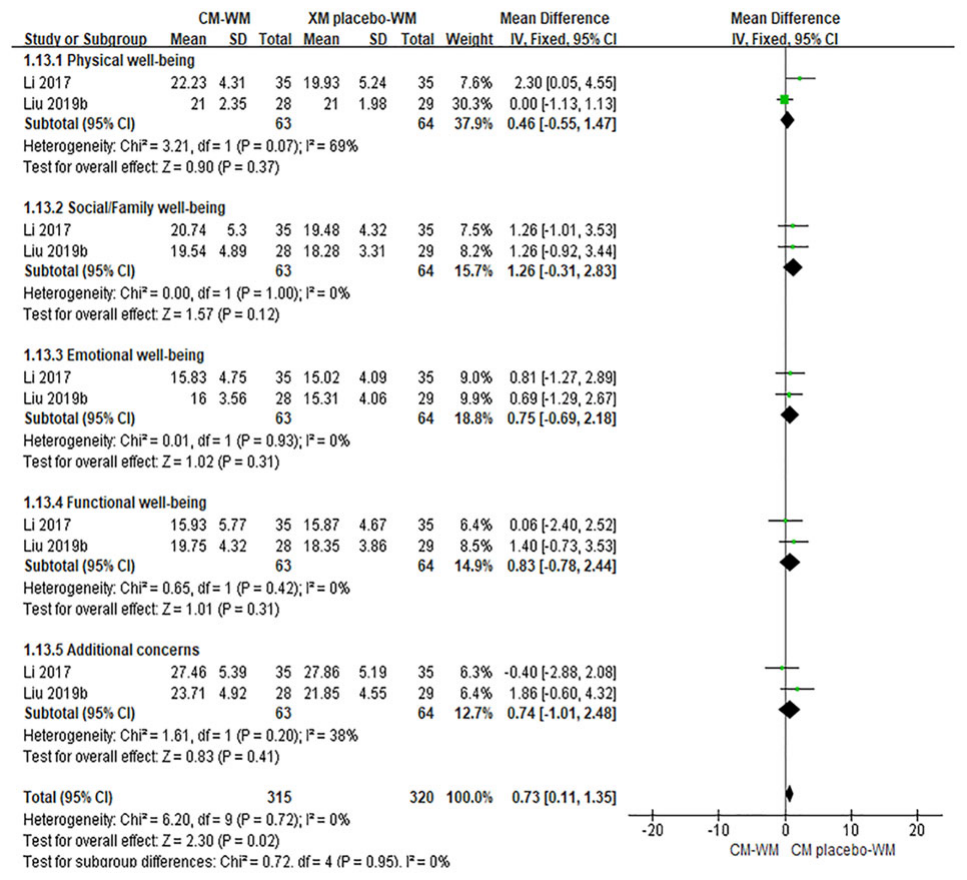
Meta-analyses on FACT-B (CM-WM vs CM placebo-WM).

**Figure 5:**
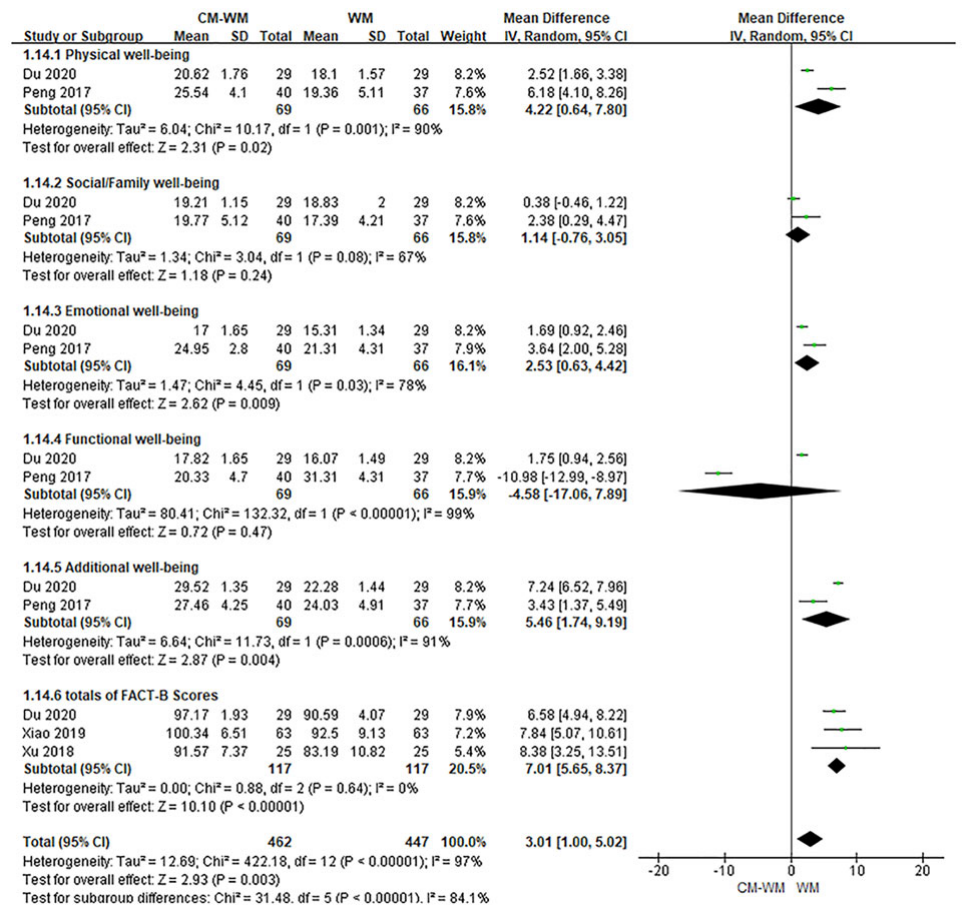
Meta-analyses on FACT-B (CM-WM vs WM).

Six trials (20, 28, 29, 31, 40, 42) mentioned the quality of life by the Functional Assessment of Cancer Therapy-Breast (FACT-B) after treatment. As indicated in the forest plot, the quality of life in two trials (28, 42) was significantly improved after receiving the CM-WM treatment compared with CM placebo-WM treatment (*P* = 0.003, MD = 0.73, 95% CI = 0.11–1.35, **Figure 4**); and in four trials was also significantly improved after receiving the CM-WM treatment compared with WM treatment (20, 29, 31, 40) (*P* = 0.003, MD = 3.01, 95% CI = 1.00–5.02, **Figure 5**). In addition, another three trials (33, 36, 44) also reported the quality of life improved significantly in CM-WM group (P <0.05). But they used different evaluation and data processing methods (QLQ-BR53, QLSBC and QOL), so the data cannot be included for this meta-analysis.

##### Pain Assessment

**Figure 6:**
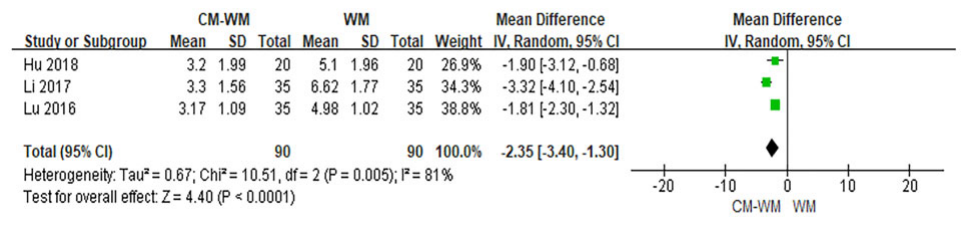
Meta-analyses on VAS.

Three trials (26, 28, 37) used Visual Analog Scale (VAS) to evaluate the pain status. As indicated in the forest plot, the mean VAS was significantly reduced in CM-WM treatment compared with WM group (P <0.001, MD = −2.35, 95% CI = −3.40 to −1.30, **Figure 6**).

##### Efficacy of TCM Symptoms

**Figure 7:**
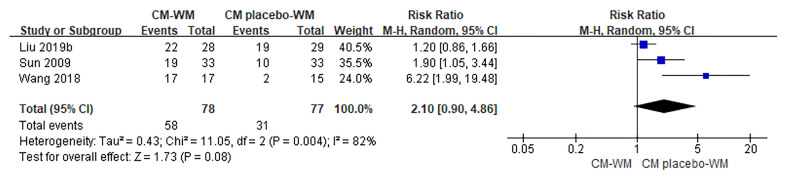
Meta-analyses on TCM Symptoms (CM-WM vs CM placebo-WM).

**Figure 8:**
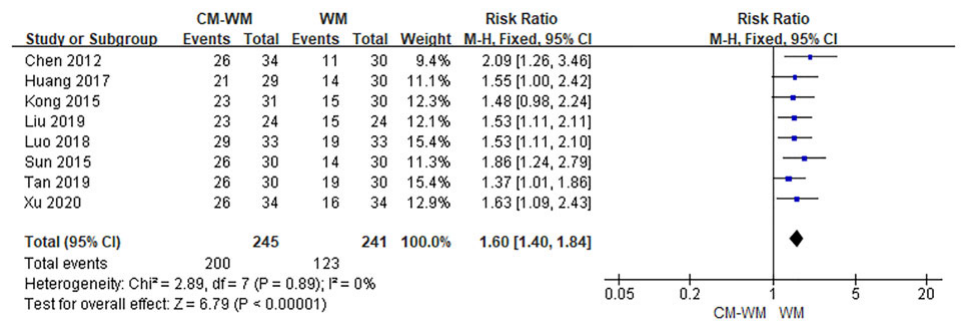
Meta-analyses on TCM Symptoms (CM-WM vs WM).

Three trials (17, 35, 42) compared the improvement of TCM symptoms between CM-WM group and CM placebo-WM group. As indicated in the forest plot, there was no significant difference between two groups (*P* = 0.08, RR = 2.10, 95% CI = 0.90–4.86; **Figure 7**). Eight trials (18, 22, 24, 30, 33, 39, 41, 44) compared the improvement of TCM symptoms between CM-WM group and CM placebo-WM group after the treatment. As indicated in the forest plot, the TCM symptoms were significantly relieved in CM-WM treatment that compared with WM group (*P <*0.0001, RR = 1.60, 95% CI = 1.40–7.84; **Figure 8**).

##### Immunological Functions

**Figure 9:**
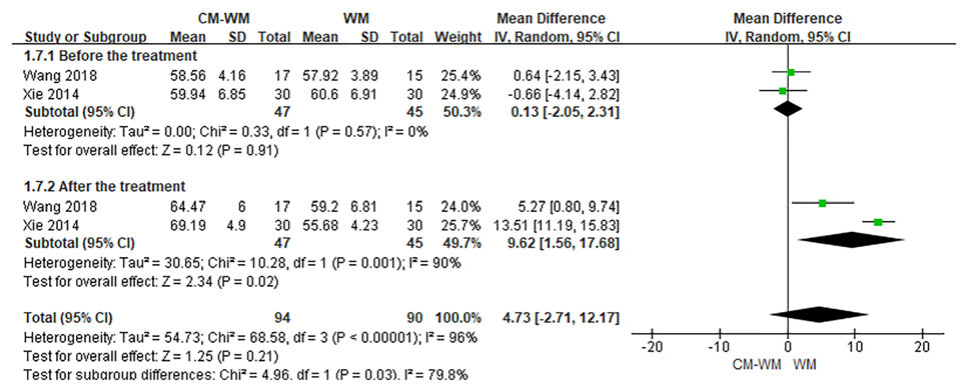
Meta-analyses on CD3.

**Figure 10:**
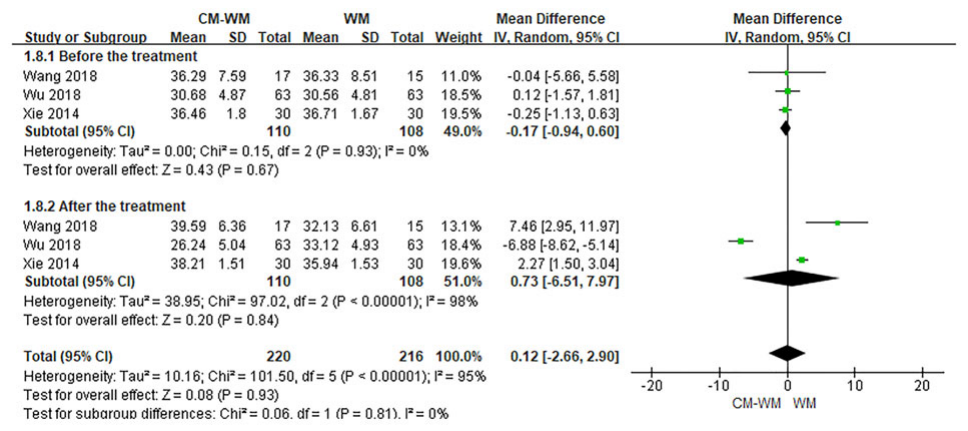
Meta-analyses on CD4.

**Figure 11:**
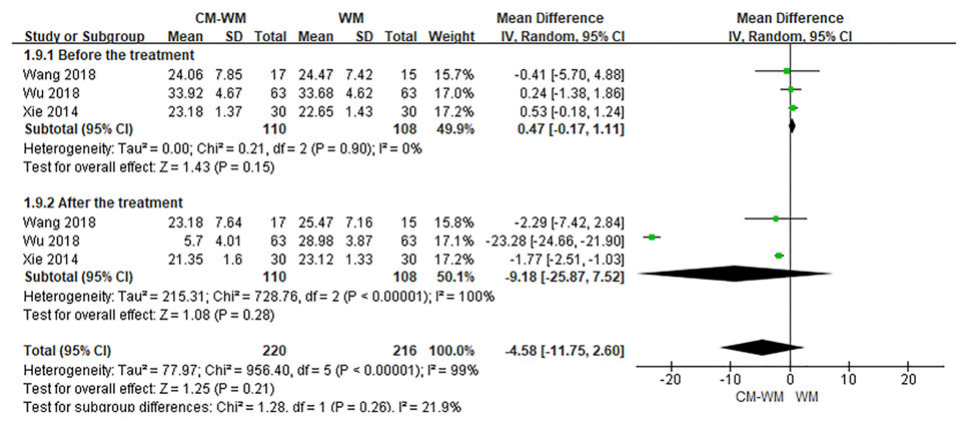
Meta-analyses on CD8.

Three trials (20, 35, 44) reported the CD3, CD4 and CD8 counts changes. But no significant differences were found in CD3, CD4 or CD8 counts before or after interventions between CM-WM group and WM group (*P* = 0.21, MD = 4.73, 95% CI = −2.71–12.17, **Figure 9**; *P* = 0.93, MD = 0.12, 95% CI = −.2.66–2.90, **Figure 10**; *P* = 0.21, MD = −4.58, 95% CI = −11.75–2.60, **Figure 11**).

##### The Serum Calcium Concentration

**Figure 12:**
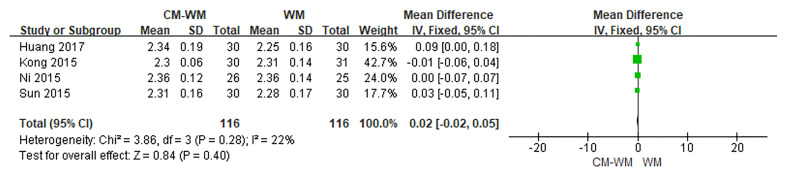
Meta-analyses on Calcium.

Four trials (22–24, 30) reported changes of the blood calcium concentrations. But no significant differences were found before or after the treatment between CM-WM group and WM group (*P* = 0.40 MD = 0.02, 95% CI = −0.02–0.05, **Figure 12**).

#### Safety

##### TCM Syndrome Scores

**Figure 13:**
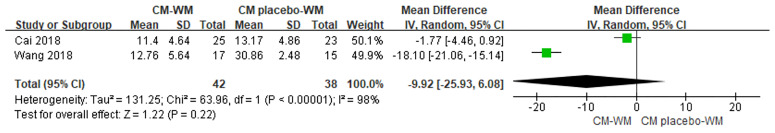
Meta-analyses on TCM syndrome sores (CM-WM vs CM placebo-WM).

**Figure 14:**
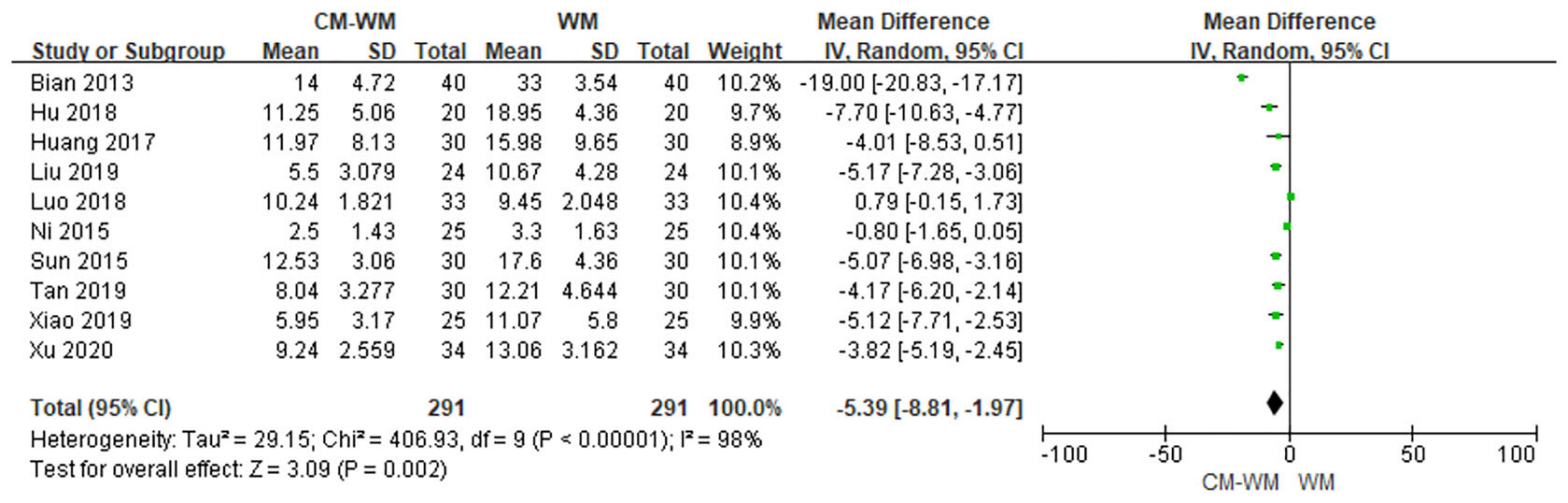
Meta-analyses on TCM syndrome scores (CM-WM vs WM group).

Two trials (33, 36) compared TCM syndrome sores after the treatment between CM-WM group and CM placebo-WM group. As indicated in the forest plot, there was no significant differences between two groups (*P* = 0.22, MD = −9.92, 95% CI = −25.93 to −6.08, **Figure 13**). Ten trials (19, 22, 23, 30, 33, 37, 39–41, 44) compared TCM syndrome sores after the treatment between CM-WM group and WM group. As indicated in the forest plot, the scores in CM-WM group was significantly lower compared with WM group (*P* = 0.002, MD = −5.39, 95% CI = −8.81 to −1.97, **Figure 14**).

##### ALP

**Figure 15:**
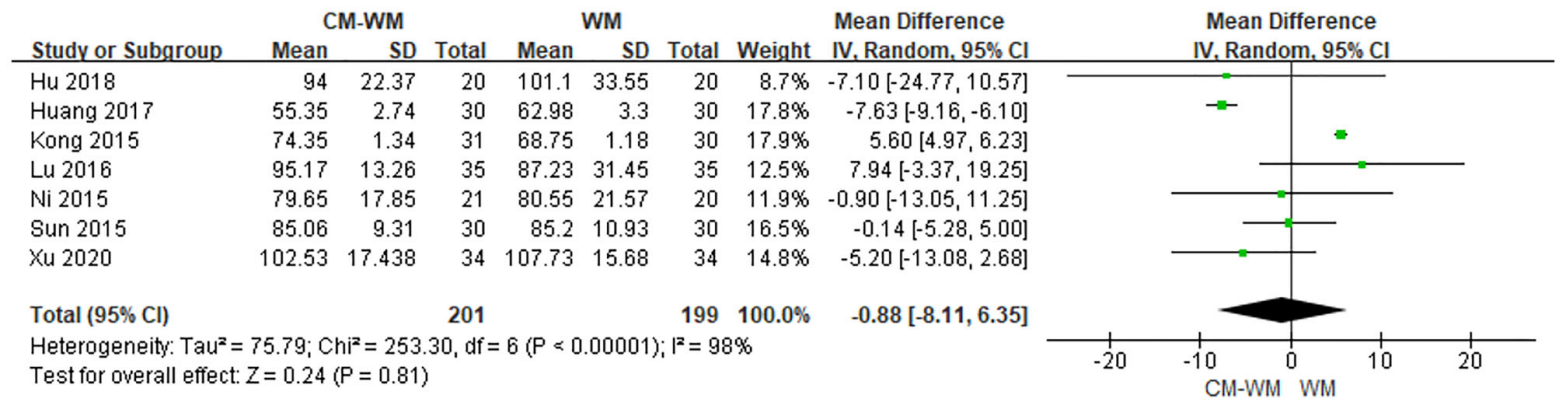
Meta-analyses on ALP.

Seven trials (22–24, 26, 30, 37, 44) tested ALP between CM-WM group and WM group after treatment. As indicated in the forest plot, no significant differences were found between these two treatments (*P* = 0.81, MD = −0.88, 95% CI = −8.11–6.35, **Figure 15**).

##### The Performance Status

**Figure 16:**
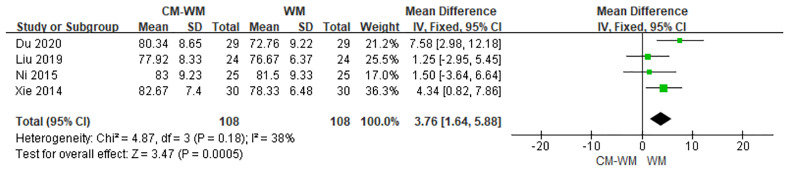
Meta-analyses on KPS.

The improvement of performance status were evaluated in four trials (20, 23, 39, 43) according to Karnofsky Performance Scale (KPS) between CM-WM group and WM group after the treatment. As indicated in the forest plot, mean KPS scores in CM-WM group were significant higher than in WM group (P = 0.0005, MD = 3.76, 95% CI = 1.64–5.88, **Figure 16**).

##### Hormone Levels

**Figure 17:**
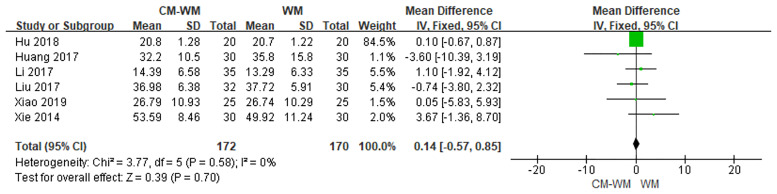
Meta-analyses on E_2_.

Serum estradiol (E_2_) after the treatment were recorded in six trials (20, 27, 28, 30, 37, 40). As indicated in the forest plot that no significant differences of estradiol level were found between CM-WM group and WM group (*P* = 0.70, MD = 0.14, 95% CI = −0.57–0.85, **Figure 17**).

##### Safety Assessments

**Figure 18:**
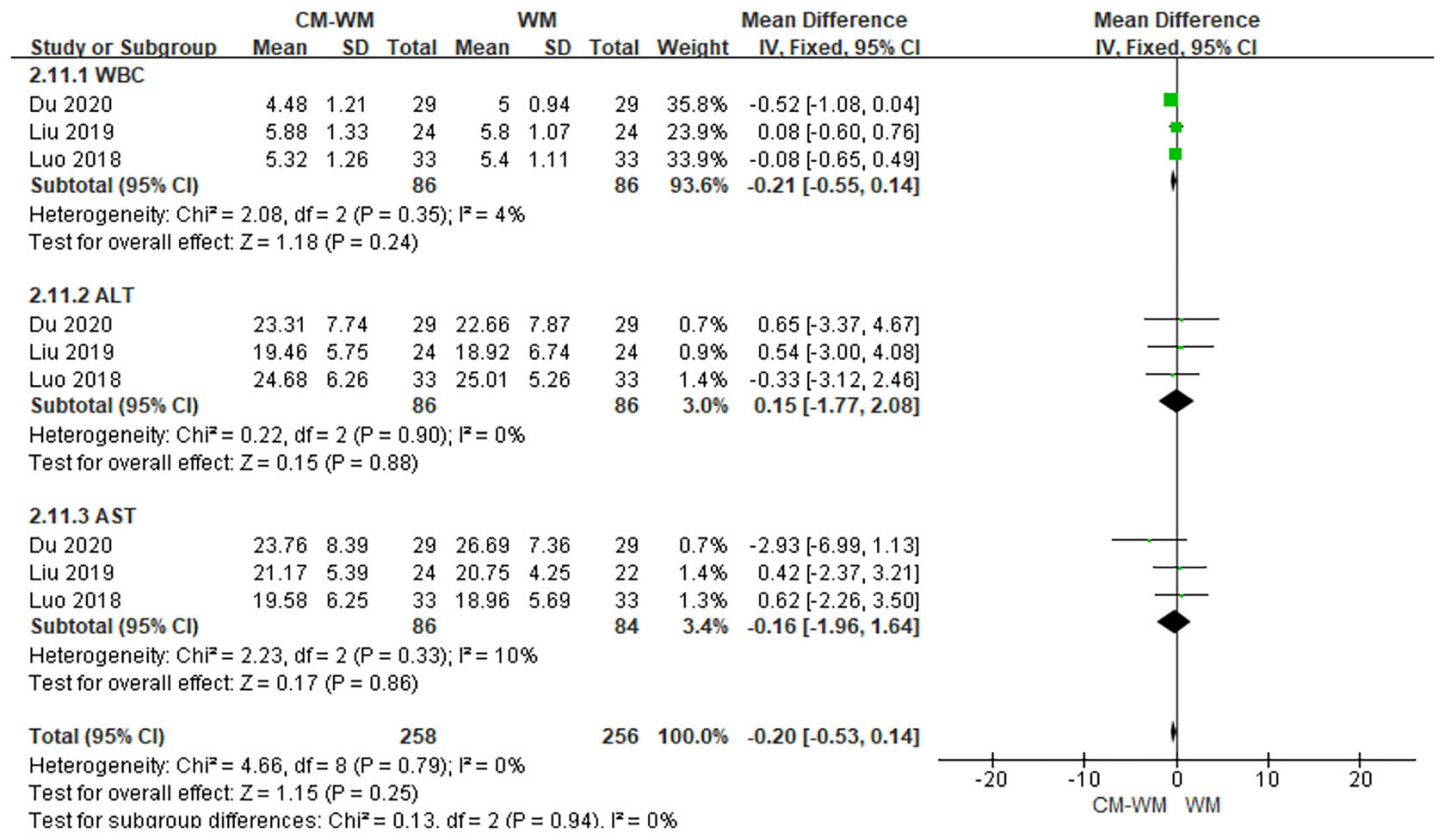
Meta-analyses on safety assessments.

Three trials (33, 39, 43) recorded the safety assessments during the treatment. As indicated in the forest plot that that no significant differences of safety assessments level were found between CM-WM group and WM group (*P* = 0.25, MD = −0.20, 95% CI = −0.53–0.14, **Figure 18**). There are eight trials mentioned the safety assessment during the trials, but the incidence was not reported. No serious adverse events were recorded in any of the studies.

## Discussion

Currently, surgery-based treatment is considered as mainstream for breast cancer (45). Endocrine therapy, in particular, is one of the common approaches to improve patients’ survival after the surgery and to prevent recurrence and metastasis (46), but it often induces various adverse reactions (47). With the development of complementary and alternative medicine, CM-WM has become an indispensable adjuvant therapy for patients with breast cancer (48–50). According to TCM theory, the adverse effects of breast cancer treatment were mostly due to the deficiency of vital energy after surgery, radiotherapy and endocrine therapy. Tonifying Qi, nourishing Blood, soothing Liver and regulating Qi, dispelling Blood stasis and detoxification, resolving Phlegm and dispersing stasis by CM are very helpful to the patients. In addition, activating blood circulation and removing blood stasis can also restore the body to a state of relative balance between Yin and Yang, which promote the recovery of disorder.

In this review, we analyzed the efficacy and safety of CM-WM as adjuvant treatment for endocrine therapy for breast cancer after surgery. The meta-analyses showed that in the comparison to WM as treatment alone, CM-WM treatment played an important role in improving the patients’ life quality, clinical symptoms such as nausea and vomiting, constipation, fatigue and the immunology function. In addition, results based on available literatures indicated that the adjunctive use of CM may reduce the endocrine therapy associated adverse events, including decreased BMD, reduced perimenopausal symptoms and impaired immune function. No severe adverse outcomes or reactions were recorded in the included studies, suggesting that CM-WM intervention was safe in treating endocrine therapy induced side effects. Bone loss is a common side effect induced by endocrine therapy. 13 trails recorded the changes in BMD, and the meta-analysis result showed that compared with WM group, patients had higher BMD in CM-WM group. It suggested that Chinese Medicine intervention significantly reduces the side effect of bone loss after endocrine therapy, which potentially reduces fragility fracture or secondary osteoporosis.

However, this review has limitations. Firstly, only five of 28 included RCTs reported blinding. Double blinding method is not feasible due to the trial setting and ethics in cancer patients. About 15 studies specifically reported the randomized method used in the study, the other 13 studies only reported a general wording”randomization”. Secondly, the sample size was not big in most included RCTs; only three studies had more than 100 participants. Last but not the least, CM formulae used in the trial might not always the same as in included clinical trials. Because according to the TCM theory, personal therapy regimen, including modifications of the individual CM in the formula and their dose, should be individually applied following the change of patients’ health conditions and TCM syndrome from time to time.

## Conclusion

CM-WM treatment has fewer adverse outcomes than using western medicines alone on breast cancer patients after reduction surgery with endocrine therapy. CM-WM treatment also has a unique superiority on improving life quality caused by adjuvant endocrine therapy. However, higher quality large-scale RCTs are needed to support the effectiveness and safety of CM-WM therapy.

## Data Availability Statement

The original contributions presented in the study are included in the article/**Supplementary Material**. Further inquiries can be directed to the corresponding authors.

## Author Contributions

LL and XF contributed conception and design of the study. LL, RW, and QS organized the databases. LL, RW, AZ, QS, QG, YL, and TC performed the statistical analysis and prepared the figures and tables. LL, XF, RW, QS, AZ, and QG wrote the first draft of the manuscript. YL, TC, and LW wrote the sections of the manuscript. CCW, PCL, and XF modified the English. All authors contributed to the article and approved the submitted version.

## Funding

This study was funded by Zhejiang Provincial Natural Science Foundation of China (LY20H180004 and LY14H240001), Key Research-Development Program of Zhejiang Province (2017C03013), National Key Research-Development Program of China (2019YFE0198800), Qianjiang Talents Fund of Zhejiang Province (QJD1602026), and Zhejiang Provincial Public Welfare Research Project (LGF19H270002).

## Conflict of Interest

The authors declare that the research was conducted in the absence of any commercial or financial relationships that could be construed as a potential conflict of interest.
