# Supplementary material for: Evidence on Efficacy and Safety of Chinese Medicines Combined Western Medicines Treatment for Breast Cancer With Endocrine Therapy
Source: Front Oncol. 2021 Jun 21;11:661925. doi: 10.3389/fonc.2021.661925 (PMC8255804; doi:10.3389/fonc.2021.661925)
Supplement: Supplementary file 1 [file DataSheet_1.docx]

| Supplementary Table: All meta-analysis | | | | | |
| --- | --- | --- | --- | --- | --- |
| Outcomes | No.of Studies | No.of Participants  (T/C) | Analysis Results (95%CI),  Overall effect P value | Heterogeneity  P value, I^2^(%) | Analytical model |
| Bone mineral density | 13 | 484/470 | MD=0.24 (0.13~0.35),  P＜0.00001 | P＜0.00001,  I^2^=91 | random-effect |
| Kupperman scales | 5 | 1132/1107 | MD=-2.35 (-2.76~-1.94),  P＜0.00001 | P＜0.00001,  I^2^=83 | random-effect |
| FACT-B | 2 | 315/320 | MD=0.73 (0.11~1.35),  P=0.02 | P=0.72,  I^2^=0 | fixed-effect |
|  | 4 | 462/447 | MD=3.01 (1.00~5.02), P=0.003 | P＜0.00001,  I^2^=97 | random-effect |
| VAS Scales | 3 | 90/90 | MD=-2.35 (-3.40~-1.30),  P<0.001 | P=0.005,  I^2^=81 | random-effect |
| Efficacy of TCM symptoms | 3 | 78/77 | RR=2.10 (0.90~4.86),  P=0.08 | P=0.004,  I^2^=82 | random-effect |
|  | 8 | 245/241 | RR=1.60 (1.40~1.84),  P<0.0001 | P=0.89,  I^2^=0 | fixed-effect |
| CD3 | 2 | 94/90 | MD=4.73 (-2.71~12.17),  P=0.21 | P＜0.00001,  I^2^=96 | random-effect |
| CD4 | 3 | 220/216 | MD=0.12(-2.66~2.90), P=0.93 | P＜0.00001,  I^2^=95 | random-effect |
| CD8 | 3 | 220/216 | MD=-4.58 (-11.75~2.60),  P=0.21 | P＜0.00001,  I^2^=99 | random-effect |
| Serum calcium concentration | 4 | 116/116 | MD=0.02 (-0.02~0.05),  P=0.40 | P=0.28,  I^2^=22 | fixed-effect |
| TCM syndrome score | 2 | 42/38 | MD=-9.92 (-25.93~-6.08),  P=0.22 | P＜0.00001,  I^2^=98 | random-effect |
|  | 10 | 291/291 | MD=-5.39 (-8.81~-1.97),  P=0.002 | P＜0.00001,  I^2^=98 | random-effec |
| ALP | 7 | 201/199 | MD=-0.88 (-8.11~6.35),  P=0.81 | P=0.81,  I^2^=98 | random-effect |
| KPS | 5 | 108/108 | MD=3.76 (1.64~5.88),  P=0.0005 | P=0.18,  I^2^=38 | fixed-effect |
| Estradiol | 6 | 172/170 | MD=0.14 (-0.57~0.85),  P=0.70 | P=0.58,  I^2^=0 | fixed-effect |
| Safety assessment | 3 | 258/225 | MD=-0.21(-0.54~0.13), P=0.28 | P=0.79,  I^2^=0 | fixed-effect |
